# Resistance gene transfer: induction of transducing phage by sub-inhibitory concentrations of antimicrobials is not correlated to induction of lytic phage

**DOI:** 10.1093/jac/dkx056

**Published:** 2017-03-20

**Authors:** Kinga I. Stanczak-Mrozek, Ken G. Laing, Jodi A. Lindsay

**Affiliations:** Institute for Infection and Immunity, St George’s, University of London, Cranmer Terrace, London SW17 0RE, UK

## Abstract

**Objectives:** Horizontal gene transfer of antimicrobial resistance (AMR) genes between clinical isolates via transduction is poorly understood. MRSA are opportunistic pathogens resistant to all classes of antimicrobial agents but currently no strains are fully drug resistant. AMR gene transfer between *Staphylococcus aureus* isolates is predominantly due to generalized transduction via endogenous bacteriophage, and recent studies have suggested transfer is elevated during host colonization. The aim was to investigate whether exposure to sub-MIC concentrations of antimicrobials triggers bacteriophage induction and/or increased efficiency of AMR gene transfer.

**Methods:** Isolates from MRSA carriers were exposed to nine antimicrobials and supernatants were compared for lytic phage particles and ability to transfer an AMR gene. A new technology, droplet digital PCR, was used to measure the concentration of genes in phage particles.

**Results:** All antibiotics tested induced lytic phage and AMR gene transduction, although the ratio of transducing particles to lytic particles differed substantially for each antibiotic. Mupirocin induced the highest ratio of transducing versus lytic particles. Gentamicin and novobiocin reduced UV-induced AMR transduction. The genes carried in phage particles correlated with AMR transfer or lytic particle activity, suggesting antimicrobials influence which DNA sequences are packaged into phage particles.

**Conclusions:** Sub-inhibitory antibiotics induce AMR gene transfer between clinical MRSA, while combination therapy with an inhibiting antibiotic could potentially alter AMR gene packaging into phage particles, reducing AMR transfer. In a continually evolving environment, pathogens have an advantage if they can transfer DNA while lowering the risk of lytic death.

## Introduction

Generalized transduction is a key mechanism of antimicrobial resistance (AMR) gene transfer between many bacteria, including the major AMR pathogen *Staphylococcus aureus*.^1^ MRSA is the most frequently identified nosocomial pathogen in hospitals in many parts of the world^2^ and the most prevalent cause of serious AMR infections.^3^ Successful hospital-associated (HA) MRSA are resistant to nearly all β-lactam, carbapenem and cephalosporin antibiotics due to the *mecA* gene. In addition, resistance to all classes of antibiotics has been described in HA-MRSA clones, although no individual isolates are resistant to all. Most of these resistances are due to genes encoded on mobile genetic elements such as plasmids, transposons and SCC*mec*.^4^

Horizontal gene transfer between *S. aureus* is predominantly due to generalized transduction via bacteriophage, and all clinical isolates have prophages in their genomes.^1^ In contrast, transformation occurs at very low efficiency and conjugative elements are found in only a small fraction of isolates.^1^^,^^5^^,^^6^ Clinical isolates of MRSA (and *S. aureus*) carry at least one prophage, and some isolates carry up to four.^5^^,^^7^ Prophage can be induced to excise from the chromosome and replicate by environmental stresses, such as UV light via the SOS system, followed by release of infectious phage particles.^8^^,^^9^ Transfer of phage DNA (transduction of phage followed by lysogeny) between colonizing *S. aureus* populations has been reported in patients with cystic fibrosis.^10^ Generalized transduction occurs when induced phage particles package host chromosomal or plasmid DNA instead of replicating phage DNA, and on cell lysis these particles deliver host DNA to new recipient *S. aureus.*^11^ Typical small rolling circle plasmids with AMR genes are reported to be packaged as concatemers in phage particles lacking phage DNA.^12^ The number of transducing particles in a lysate or supernatant is usually reported as a fraction of normal lytic phage particles, and is generally low.^11^

Clinical MRSA populations are challenging to genetically manipulate in the laboratory, in part due to restriction modification systems.^1^^,^^13^ However, in the host they can acquire and lose different mobile genetic elements with different AMR genes resulting in mixed populations within a single hospital or outbreak.^4^^,^^14–16^ AMR provides a selective advantage for HA-MRSA^14^^,^^17^ and the spread of the successful HA-MRSA CC22 SCC*mecIV* clone has been associated with increased gain and loss of multiple AMR.^14^^,^^18^ More recently, in piglet studies we have shown an unexpectedly high frequency of AMR gene transfer between *S. aureus* populations during experimental co-colonization.^19^ We then showed that MRSA colonized patients harboured variant populations differing only in AMR genes, as well as free bacteriophage capable of generalized transduction.^20^ This suggests gene transfer of AMR genes during colonization in the natural habitat may be much higher than expected. Sub-inhibitory concentrations of antibiotics induce bacteriophage excision and replication^8^^,^^21^^,^^22^ and antibiotics such as β-lactams, trimethoprim and ciprofloxacin have been shown to enhance phage induction *in vitro* and to increase the ability to transfer virulence genes between laboratory strains of *S. aureus*.^8^^,^^23^ Thirty percent of hospitalized patients receive antibiotics,^3^^,^^24^ directly or indirectly acting on colonizing MRSA populations that are the reservoir of subsequent infecting isolates. It is not known if this exposure affects the ability of colonizing MRSA populations to transfer AMR genes.

In this study, we aimed to investigate whether sub-MIC concentrations of different antibiotics trigger prophage induction from MRSA carriage isolates, leading to lysis and/or increasing efficiency of transfer of resistance genes. We found that all antimicrobials induced transfer, although this was not correlated with their ability to induce lytic phage particles.

## Materials and methods

### Strains and antibiotics

Two colonizing MRSA isolates, 19A and 19B,^20^ were selected as donor and recipient strains, respectively, for phage preparation and transduction. Both isolates have been whole genome sequenced and belong to the dominant HA-MRSA clone in the UK, CC22 SCC*mecIV*, and carry Φ1, Φ2 and Φ3 phages and an SaPI4 element.^20^ 19A is positive for *ermC* and a *rep10* plasmid, and both are negative for conjugative transfer genes (*tra*) (see Table S1, available as Supplementary data at *JAC* Online). RN4220 is restriction-modification negative and was used as a control recipient.^25^

Antibiotics, apart from ciprofloxacin, were obtained from Sigma–Aldrich Ltd. Ciprofloxacin was purchased from Stratech Scientific Limited as a 10 mg/mL solution ready to use. MICs of antibiotics were determined by the standard growth microdilution method with Iso-Sensitest broth (Oxoid) with a bacterial inoculum of 1 × 10^6^ cfu/mL and determined as the lowest concentration of an antimicrobial agent that inhibited the visible growth after overnight incubation at 37°C (Table S2).

### Exposure to antibiotics

Donor 19A was grown in 7 mL of LK broth (1% tryptone/0.5% yeast extract/0.7% KCl; all purchased from Sigma–Aldrich Ltd) at 37°C with shaking until log phase (OD=0.5–1 at 600 nm). Bacteria were spun down and resuspended in 7 mL of phage buffer (50 mM Tris-HCl, pH 7.8/100 mM NaCl/1 mM MgSO_4_/4 mM CaCl_2_/1 g of gelatin; Sigma–Aldrich Ltd). Antibiotics were added (Table 1), alone or in combination, followed by 7 mL of sterilized brain heart infusion broth (BHIB; Sigma–Aldrich Ltd). Samples were vortexed gently and incubated at room temperature for 10 min. After overnight incubation at 30–32°C with gentle shaking, samples were centrifuged for 10 min at 3000 **g** and filtered through a 0.22 μm filter (Millipore).

**Table 1:** Sub-MIC concentrations of antibiotics used in this study

Antibiotic	Sub-MIC (mg/L)
Erythromycin	30
Trimethoprim	1
Ampicillin	30
Mupirocin	1
Tetracycline	1
Novobiocin	0.25
Cefoxitin	30
Gentamicin	30
Ciprofloxacin	30

The impact of sub-MIC concentrations of antibiotics on UV light-induced phage was tested by a similar method. One mL of the LK broth log-phase culture was resuspended in 1 mL of phage buffer, pipetted into a sterile Petri dish and then 6 mL of phage buffer was added. The Petri dish was placed under the UV light source with the lid off for 30 s. Exposed bacteria were transferred with a pipette into a 50 mL Falcon tube followed by addition of antibiotic (Table 1). Controls with bacteria exposed to UV light only or selected antibiotic only as well as those not exposed at all were always included. Seven mL of BHIB was added, and incubated overnight as before.

### Titration of lytic phage

Titration of phage preparations was performed as described previously^20^ using RN4220 as an indicator strain. Briefly, 200 μL of phage preparation was mixed with 200 μL of recipient cells (RN4220) in log phase (OD=1 at 600 nm) followed by addition of 30 μL of 1 M CaCl_2_. Samples were left at room temperature (RT) for 15 min, mixed with ∼7 mL of molten top agar and poured over solidified phage bottom agar plates. Phage agar was prepared by mixing 3 g/L yeast extract (Sigma–Aldrich Ltd), 3 g/L casamino acids (Fisher-Scientific), 5.9 g/L NaCl (Sigma–Aldrich Ltd) and either 10 g/L agar (Sigma–Aldrich Ltd) (bottom agar) or 3.3 g/L agar (top agar). Plates were incubated at 30°C for 24 h and the number of plaques counted to calculate pfu/mL.

### Generalized transduction

Generalized transduction was performed as described previously.^20^ Recipient bacteria were grown in BHIB overnight at 37°C with shaking, centrifuged for 10 min at 3000 **g** and resuspended in 1 mL of LK broth. Recipient cells were mixed with 1 mL of LK broth and 0.5 mL of phage preparation and CaCl_2_ was added to a final concentration of 8 mM followed by 20 μg/mL DNase (Promega). Samples were incubated at 31°C for 45 min. Control tubes with recipient cells only and phage preparation only were also prepared. After incubation, ice-cold 0.02 M sodium citrate was added (Honeywell International Inc.) to a final concentration of 15 mM and samples centrifuged at 3000 **g** for 10 min. The supernatant was decanted, the pellet resuspended in 1 mL of ice-cold 0.02 M sodium citrate and left on ice for at least 2 h. Samples were spread on LK plates prepared by mixing LK broth components with 5 g of bacteriological agar supplemented with 0.05% sodium citrate and 0.15 mg/L erythromycin and incubated at 37 °C for 60 min, then overlaid with 4–5 mL of LK top agar supplemented with 30 mg/L erythromycin. Plates were incubated for 48 h at 37°C. The number of transductant cells was counted and expressed as the number of transductant cells per mL instead of the commonly used frequency of transductants per pfu, as not all particles will carry virulent phage genome and cause lysis. Selected colonies of transductants were picked and passaged on mannitol salt agar to confirm they were *S. aureus* and on brain heart infusion agar with 30 mg/L erythromycin. DNA from up to 20 transductant colonies per antibiotic was extracted and the presence of *ermC* confirmed by PCR.

### Isolation and purification of genomic DNA from phage particles

Five hundred mL of phage preparation was clarified by filter sterilization through a stericup vacuum filtration system with a 0.22 μm filter (Millipore). Five mL of 5 mM MgSO_4_ was added followed by addition of 5 μg/mL DNase I (Roche) and 10 μg/mL RNase A (Sigma–Aldrich Ltd), followed by incubation for 1 h at 37°C. Solid PEG 8000 (Promega) was added to a final concentration of 30% and dissolved by slow stirring at 4°C for 48 h. The solution was centrifuged at 3000 **g** for 45 min and the pellet resuspended in 3 mL of SM buffer (100 mM NaCl/8 mM MgSO_4_·7H_2_O/50 mM Tris-Cl, pH 7.5; all from Sigma–Aldrich Ltd) and left for 30 min at RT. Proteinase K (Qiagen) was added to a final concentration of 50 μg/mL, SDS (Sigma–Aldrich Ltd) was added to a final concentration of 0.5% and 0.5 M EDTA (pH 8.0) (Sigma–Aldrich Ltd) was added to a final concentration of 20 mM, followed by incubation at 56°C for 1 h and cooling to RT. An equal volume of solution containing phenol, chloroform and isoamyl alcohol in a 25:24:1 ratio (Sigma–Aldrich Ltd) was added, mixed gently and centrifuged at 12 000 **g** for 10 min. The aqueous phase was recovered and an equal volume of chloroform was added then centrifuged at 12 000 **g** for 10 min. The aqueous phase was pipetted into a clean microfuge tube and washed three times with chloroform. One mL of 100% ethanol was added and the sample was left at −20°C overnight, spun at 12 000 **g** for 20 min and the pellet washed three times with 1 mL of 70% ethanol and dissolved in 50 μL of distilled water. The DNA was purified by using the Wizard SV gel and PCR clean-up system (Promega) according to the manufacturer's instructions. Nanodrop and gel electrophoresis were used to quantify DNA.

### Droplet digital PCR (ddPCR) assay

The QX100 ddPCR system from Bio-Rad was used. Four genes were selected for ddPCR analysis. The *ermC* erythromycin resistance gene located on the *rep10* plasmid, the staphylococcal nuclease gene *nuc* located on the bacterial chromosome and two genes encoding bacteriophage integrase genes for Φ1 and Φ2. Primer and probe sequences are provided in Table S3 and were used in paired combinations as either Φ2 and *ermC* or *nuc* and Φ1. Φ2 and *nuc* probes were labelled with 5′ FAM and *ermC* and Φ1 were labelled with 5′ Yakima Yellow (using the VIC HEX channel on the QX100). A 20× concentrated primer probe mix for ddPCR was prepared by mixing 12 μL of each of two selected forward primers (600 nM), 6 μL of each of reverse primers (600 nM for *nuc* gene and 300 nM for the other genes), 4 μL of each probe and made up to 100 μL of distilled water, then aliquoted and kept at –20°C. The master mix for ddPCR for each sample assay was prepared by mixing 10 μL of a 2× ddPCR Supermix for probes (Bio-Rad), 1 μL of primer probe mix (prepared earlier), 1 μL of distilled water and 8 μL of DNA to a final reaction volume of 20 μL. The entire reaction mixture was loaded into one of eight sample wells of a plastic microfluidic cartridge (Bio-Rad); 70 μL of droplet generation oil (Bio-Rad) was loaded into the corresponding oil reservoir (all eight positions in the cartridge were filled) and placed in the droplet generator (Bio-Rad). The droplets generated from each sample were transferred to a 96-well standard profile 0.2 mL PCR plate (Eppendorf) and pipetted by aspirating and dispensing the 90 μL volume over an 8 s duration to avoid damaging the emulsion. The plate was sealed with easy-pierce foil. PCRs were performed using the following cycle conditions: 95°C denaturation for 10 min, followed by 40 cycles of 94°C for 30 s and 58°C for 60 s, 1 cycle of 98°C for 10 min. After amplification, the plate was placed in the droplet reader, which analysed each droplet individually using a two-colour optical detection system (FAM and Yakima Yellow) and identified which droplets contain the respective target DNA sequences and which did not, according to the fluorescence amplitude compared with a baseline identified by a known negative control performed simultaneously. Samples containing amplified product with bright fluorescence relative to the baseline were considered as positive, whereas droplets with little or no fluorescence formed the sample baseline and were called negative. Data were analysed with QuantaLife^®^ analysis software, which calculated the fraction of positive droplets and fitted them to a Poisson distribution to determine the starting concentration of the target sequence in units of copies/μL input from the sample.

### Statistical analysis

Differences in phage titres or transductants/mL were analysed by unpaired two-tailed Student's *t*-test with Welch's correction. Variation in copy number of the target genes per μL detected by ddPCR was analysed using two-way ANOVA. *P* < 0.05 was considered statistically significant. Linear regression was applied to assess the correlation between the gene content of phage particles and pfu/mL or number of transductant cells/mL.

## Results

### Sub-inhibitory antibiotics induce transducing and lytic particles in different quantities and ratios

**Figure 1:**
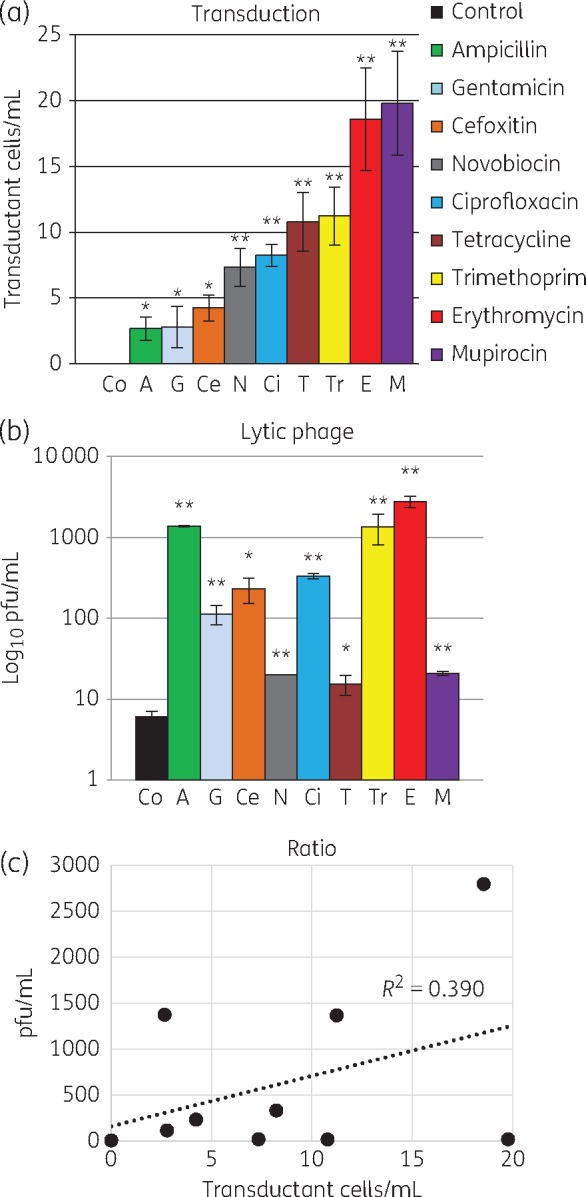
Sub-inhibitory antibiotics induce lytic and transducing particles in different ratios. (a) Transduction of *ermC* (using phage preparations treated with antibiotics) to recipient cells was significantly higher than without antibiotics (no transductants detected). (b) Lytic phage counted on RN4220 from the same phage preparations was also significantly higher than without antibiotics. (a and b) Mean of at least three experiments in triplicate (±SD). *P <* *0.05, **P <* *0.01. (c) Correlation between transducing particles and lytic phage for each phage preparation was 0.39, indicating different antibiotics induce different ratios of each phage particle. This figure appears in colour in the online version of *JAC* and in black and white in the print version of *JAC*.

Donor cell cultures exposed to sub-MIC of all the tested antibiotics generated transducing phage capable of transferring the *ermC* gene to recipient cells. In contrast, donor cell cultures not exposed to antibiotics did not generate phage particles capable of transducing the *ermC* gene at detectable levels (Figure 1a). The lack of detectable transductants in an unexposed control suggests that transduction needs to be stimulated, e.g. by the presence of antibiotic, or the number of transductants will be under the detection limit of the assay. The donor bacterium 19A was resistant to erythromycin, ampicillin, cefoxitin, gentamicin and ciprofloxacin, but exposure to these antibiotics did not correlate with increased transduction (Figure 1a).

In the absence of antibiotics, lytic phage was produced. However, sub-MIC concentrations of all the tested antibiotics induced higher levels of lytic phage (Figure 1b), indicating that antibiotic stimulation enhances phage induction and lytic phage production. The phage preparations contained a mixture of phage particles, including some capable of *ermC* gene transfer and others capable of lysis, and the ratio of these two particle types differed in response to each antibiotic (Figure 1c). Therefore, sub-inhibitory concentrations of mupirocin and erythromycin induced the most AMR gene transfer, while ampicillin, trimethoprim and erythromycin induced the most lytic phage. The ratio of transducing phage to lytic phage was highest for mupirocin, tetracycline and novobiocin, and lowest for erythromycin, ampicillin and trimethoprim (Table S4).

**Figure 2:**
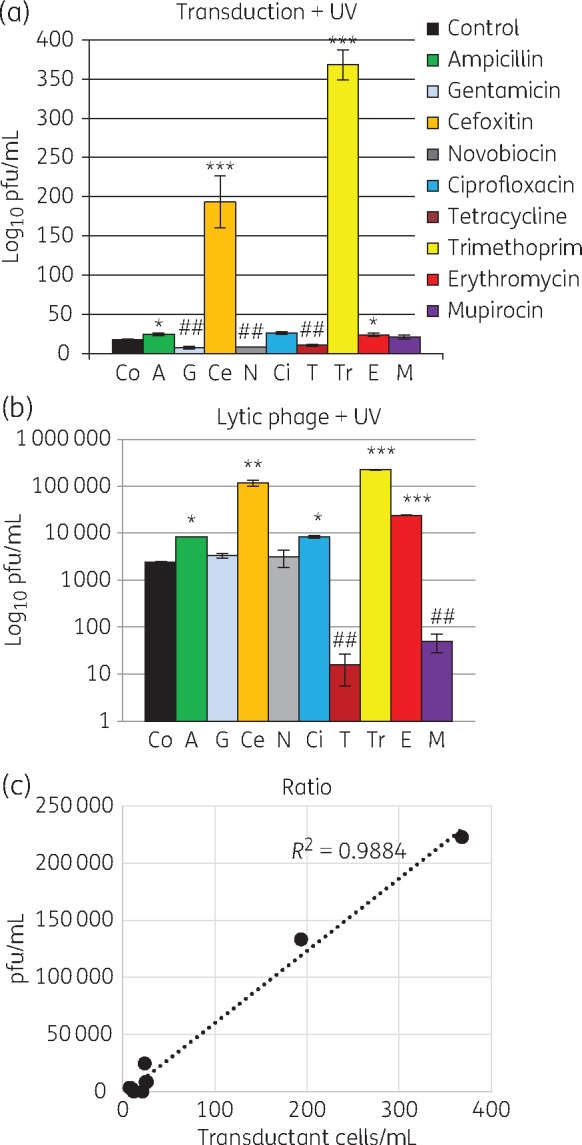
Sub-inhibitory concentrations of antibiotics combined with UV light induce lytic and transducing particles in different ratios. (a) Transduction of *ermC* (using phage preparations treated with antibiotics and UV light) to recipient cells was significantly enhanced by cefoxitin and trimethoprim, and significantly lowered by gentamicin, novobiocin and tetracycline. (b) Lytic phage counted on RN4220 was higher than UV alone when exposed to cefoxitin and trimethoprim as well as ampicillin, ciprofloxacin and erythromycin. Lytic phage production was inhibited when exposed to tetracycline and mupirocin. (a and b) Mean of at least three experiments in triplicate (±SD). *P <* *0.05, **P <* *0.01, ***P <* *0.001, a double hash denotes reduction (^##^*P *<* *0.01). (c) Correlation between transducing particles and lytic phage for each donor cell lysate was 0.99, indicating that under UV light stress, the ratio of transducing and lytic phage particle is not dependent on the antibiotic tested. This figure appears in colour in the online version of *JAC* and in black and white in the print version of *JAC*.

Stresses such as UV light are known to stimulate the SOS response and induce lytic and transducing phage particles. To determine if this effect was synergistic or antagonistic with sub-inhibitory antibiotics, the two stresses were combined. UV light dramatically increased the transducing potential when combined with cefoxitin or trimethoprim (Figure 2a). In contrast, tetracycline, novobiocin and gentamicin inhibited the effect of UV light on transduction (Figure 2a). UV light increased the number of lytic phage particles, and this was increased again when combined with cefoxitin, trimethoprim, erythromycin, ampicillin and ciprofloxacin (Figure 2b). Tetracycline and mupirocin blocked the induction of lytic particles by UV light (Figure 2b). UV light increased lytic phage particles when combined with most of the antibiotics tested, but a decrease was detected when UV light was combined with mupirocin or tetracycline (Figure 2b). UV light increased or decreased the total concentration of phage particles, such that the ratio of transducing and lytic phage particles was relatively conserved in the different lysates (Figure 2c). The data indicate that UV light acts independently of antibiotics to affect total phage particle number.

### Reduction of AMR gene transfer by sub-inhibitory antibiotics

**Figure 3:**
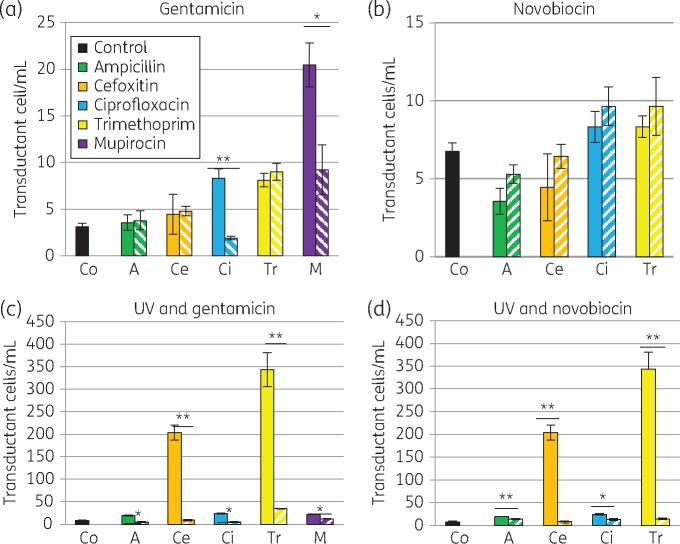
Gentamicin and novobiocin combined with sub-MIC antibiotics can reduce *ermC* transduction. Combination of gentamicin (a and c) or novobiocin (b and d) with sub-inhibitory combinations of antibiotics, without (a and b) or with (c and d) UV light. Combinations of antibiotics are indicated by hatching. Gentamicin reduced the transfer induced by ciprofloxacin and mupirocin. In the presence of UV light, both gentamicin and novobiocin reduced the high-level transfer induced by cefoxitin and trimethoprim, as well as reducing the transfer induced by all the remaining antibiotics tested. Mean of at least three experiments in triplicate (±SD). *P <* *0.05, **P <* *0.01, ***P <* *0.001. Lytic phage was also inhibited (Figure S1). This figure appears in colour in the online version of *JAC* and in black and white in the print version of *JAC*.

Novobiocin and gentamicin were two antibiotics that reduced the number of transducing particles induced by UV light (Figure 2a) and were further tested for their ability to inhibit phage particle induction when combined with other sub-inhibitory antibiotics. Gentamicin reduced transduction of *ermC* in combination with mupirocin or ciprofloxacin (Figure 3a). In combination with other antibiotics, novobiocin did not reduce transduction of *ermC* (Figure 3a), although it did reduce lytic phage (Figure S1). In contrast, both novobiocin and gentamicin very effectively reduced the high level of UV-induced transduction in the presence of trimethoprim or cefoxitin (Figure 3c and d), as well as reducing the effects of ampicillin, ciprofloxacin or mupirocin.

### DNA content of phage particles correlates with transducing and lytic activity

**Figure 4:**
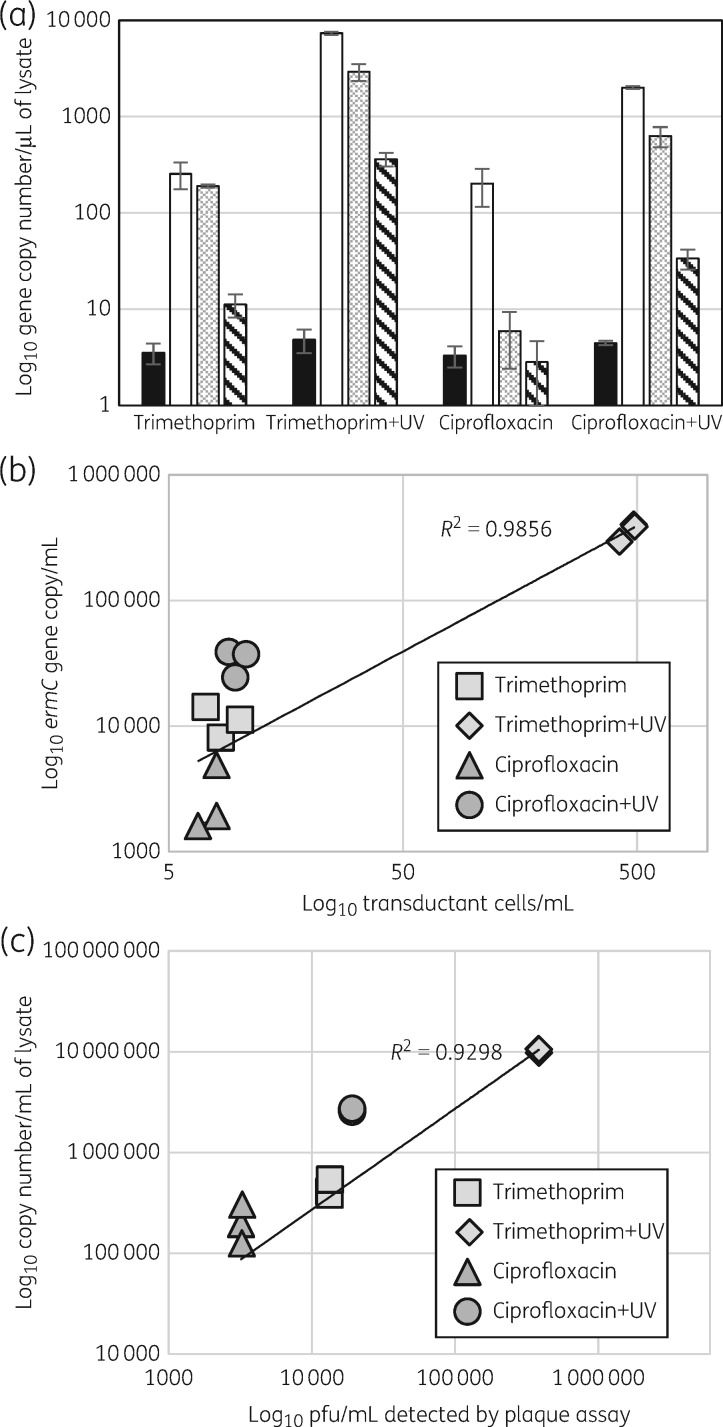
Copy number of *ermC* and phage genes in phage particles correlates with transduction and lytic activity. (a) ddPCR was used to measure the copy number of the chromosomal gene *nuc* (black), phage genes represented by Φ1*int* (white) and Φ2*int* (grey patterned) and plasmid-borne *ermC* (hatched) in purified phage particles generated by exposure to trimethoprim or ciprofloxacin with or without UV light. *ermC*, Φ1 and Φ2 copy numbers were significantly different (*P *<* *0.01) between all tested lysates apart from Φ1 exposed to ciprofloxacin and trimethoprim. The copy number of the *nuc* gene was significantly lower in comparison with *ermC* (*P *<* *0.01) and phage genes (Φ1*int* and Φ2*int*) in all lysates (*P *<* *0.001) and did not differ between lysates. Bars represent mean values of three experiments with three replicates (±SD). (b) Correlation between *ermC* concentration and transduction of erythromycin resistance. (c) Correlation between the concentration of Φ1*int* and Φ2*int* (summed) and lytic activity. Each point represents the mean of duplicate testing of three different lysates.

Phage preparations exposed to either trimethoprim or ciprofloxacin with or without UV light were compared and the concentration of *ermC*, *nuc*, Φ1*int* and Φ2*int* in the purified phage particles measured by ddPCR (Figure 4a). There was correlation between the copy number of *ermC* and the transducing ability of the lysate (Figure 4b) and correlation between the copy number of Φ1*int* and Φ2*int* genes and the lytic activity of the lysate (Figure 4c). The data indicate that the packaging step, which determines which DNA sequences are incorporated into the phage particle, plays a key role in determining the resultant transducing and lytic activity of the phage preparations.

There was no statistically significant difference in the *nuc* gene copy number between phage lysates but it was significantly lower compared with the copy number of the *ermC* gene (*P* < 0.01) and integrase genes in all samples (*P* < 0.001) (Figure 4a). This result indicates that induced phage particles package genes located on the core genome at lower frequency than phage and the plasmid *ermC* genes.

## Discussion

This study that shows sub-MIC concentrations of a range of antibiotics can stimulate the transfer of resistance genes between MRSA strains isolated from human carriers, as well as the induction of lytic phage. The natural habitat of MRSA is the nares and mucous membranes of high-risk patients, including those in frequent contact with healthcare and antimicrobial usage. Recent studies have indicated that MRSA-colonized patients often carry populations that vary in their carriage of AMR genes, likely due to generalized transduction, including sub-populations of highly resistant isolates that are not routinely detected and may act as a reservoir for AMR gene spread.

Each antibiotic tested in this study had a unique effect on the number of phage particles induced and the ratio of transducing to lytic phage particles. This could be explained by their differing mechanisms of action, although variations between β-lactams such as ampicillin and cefoxitin were seen. Importantly, all antibiotics induced AMR transfer.

Gene transfer occurs because, during phage induction, copies of the phage genome excise from the bacterial chromosome and new phage particles are made. While most particles package phage DNA, sometimes sections of chromosomal or plasmid DNA are packaged during phage assembly. These phage particles have the ability to attach to bacteria and inject the packaged DNA. Small plasmids such as those encoding AMR genes can be transduced on their own and these replicate as plasmids in the transduced recipient.

As phages are currently considered the main agents of horizontal gene transfer in *S. aureus* and MRSA,^1^ and the amount of antibiotics prescribed to hospitalized patients increases every year,^3^ the enhanced induction of potential vectors of resistance determinants could further increase the genetic exchange between MRSA strains and lead to the evolution of increasingly resistant strains. As we showed that antibiotic-inducible phages are capable of effective transduction this could possibly explain why HA-MRSA clones in the UK have successively acquired resistance to more antibiotics during the last decade.^14^ The results obtained in this study seem to support this hypothesis. Consequently, it appears that in addition to their beneficial properties, antibiotics could have collateral effects on colonizing bacteria, conceivably by enhancing the chance to survive during host colonization. Of further concern is the fact that phage lysates are known to be stable for many years in the laboratory. Indeed, host bacterial DNA encapsulated within a phage head is well protected from unfavourable environmental conditions, e.g. attack from nucleases, and can therefore survive until a suitable recipient appears.^26^

Mupirocin exposure induced the highest concentration of particles capable of transduction of *ermC* but low levels of lytic phage in the MRSA CC22 SCC*mecIV* isolate (Figure 1). This clonal type accounts for 75% of HA-MRSA in the UK.^14^ On admission to hospital, all patients in the UK are screened for MRSA in the nares, and those found to be positive are decolonized with mupirocin ointment and chlorhexidine bodywash. Therefore, mupirocin is likely the most prevalent antimicrobial to which MRSA CC22 SCC*mecIV* populations in their natural habitat are exposed. Despite exposure, phenotypic mupirocin resistance is rare.^14^ Mupirocin is effective at decreasing MRSA loads in the nares but re-colonization with the same strain after a period of weeks is common,^27^ suggesting a minor population of MRSA is maintained in the nose after treatment and when mupirocin levels have declined. We speculate that MRSA populations recovering after decolonization may have a selective advantage if they can spread genes horizontally and adapt while minimizing risk of death due to phage lysis.

In laboratory strains, antibiotics and UV light are known to activate the SOS response^8^^,^^9^^,^^28^^,^^29^ and trigger staphylococcal prophage induction, and subsequently the replication and high-frequency transfer of the staphylococcal pathogenicity islands.^23^^,^^30^ Our data show that UV light dramatically induced phage particle production in the presence of some antibiotics, but only increased AMR gene transfer in the presence of cefoxitin and trimethoprim. Indeed, the complex and variable response to each antibiotic, when combined with UV light, showed that the factors controlling phage induction and AMR gene transfer will not be ascribable to simply an SOS response.

Gentamicin and novobiocin both dramatically reduced UV-induced AMR gene transfer. Gentamicin also reduced ciprofloxacin- and mupirocin-induced AMR gene transfer (Figure 3a). Ciprofloxacin is not used for treating MRSA infections, but HA-MRSA are typically resistant and a decline in hospital prescribing has been associated with a decline in nosocomial MRSA incidence in the UK, including of the MRSA CC22 SCC*mecIV* clone.^14^ Gentamicin is used routinely in surgical prophylaxis in the UK, and may have a benefit in reducing AMR gene transfer amongst clinical isolates. Antibiotic combinations in patients may impact on colonizing and evolving MRSA populations.

Novobiocin is not widely used in human medicine as an antibiotic, but has previously been shown to reduce the SOS response induced by ciprofloxacin, decreasing the number of induced phages and potentially reducing gene transfer.^31^ In this study, novobiocin reduced AMR gene transfer induced by the combination of ciprofloxacin and UV light. Schröder *et al.*^31^ utilized the *S. aureus* laboratory isolate NCTC 8325 derived from strains circulating in the 1950s for their studies and moved a chromosomal marker, whereas we chose to investigate a contemporary nosocomial MDR MRSA in hospitals and moved a plasmid marker. Recently evolving MRSA strains have likely adapted to exposure to a range of antibiotics.

It was expected that lytic phage induction would be correlated with phage particles capable of transducing AMR genes, and indeed gene transfer frequency is often expressed as a ratio of transductants to pfu.^32^ Surprisingly, our comparison of inducing and transducing ability of lysates generated by antibiotic exposure revealed no correlation (Figure 1c). Therefore, the ratio of virulent and transducing particles formed after exposure to each antibiotic varied. To investigate if this was associated with the DNA content of the phage particles, we used a new technology, ddPCR. This acts like a traditional PCR with fluorescent tags FAM and Yakima Yellow; however, each reaction mixture is vortexed into thousands of micelles, and the fluorescent output measured by streaming individual droplets past a two-colour optical detection system. Using a mathematical algorithm, the concentration of each gene in the purified phage particle DNA can be measured. This approach was able to show a good correlation between gene content and phenotypic activity of the phage preparation. It also confirmed that the *ermC* carried on a plasmid is packaged at higher frequency than a chromosomal marker (*nuc*) and at lower frequency than phage DNA. This is likely to be due in part to the relative copy number of these genes in the host cell. Importantly, it confirmed that differences in AMR gene transfer correlated with different concentrations of *ermC* gene copy number in phage preparations (Figure 4a and b and Figure S2). DNA is packaged into phage particles via the terminus protein,^12^^,^^33^ which attaches to DNA and guides it into the newly assembled phage particle through the tail structure and into the phage head. If antibiotics influence this step, it could be at the level of DNA replication or through specific interaction with the packaging process.

The varying ratios of lytic and transducing particles detected were unexpected. Our data also show that different phages may be induced by different antibiotics. During colonization with variant populations of MRSA differing in AMR genes, there may be an advantage to the bacteria that can transfer and receive DNA while at the same time lowering the risk of lytic death. Exposure to sub-inhibitory concentrations of antimicrobials might be an appropriate signal to enhance gene exchange. Furthermore, the response of resistant bacteria to high levels of antibiotic that are sub-inhibitory has generally not been explored. Further work on the mechanisms controlling this transfer is warranted, and to identify which phages are preferentially induced and by which antibiotics.

This study has limitations. Only one selected resistance gene, *ermC*, was tested, and other AMR genes and MRSA isolates may transduce with differing frequency. Similarly, the phage carried in MRSA 19A may respond to antibiotics and UV light differently to phage carried by other *S. aureus*. Other elements such as SaPIs may also interfere with induction.^34^ The resistance profile of MRSA 19A is typical for HA-MRSA, but resistance gene carriage is likely also to interfere with the response to antimicrobials.

In conclusion, this study shows that antibiotics used clinically can increase phage mobilization and the transduction of AMR genes between clinical MRSA. All tested antibiotics induced lytic phages and transducing phages; however, the ratio differed for each antibiotic or combination antibiotic with UV light, likely due to alterations in the packaging of DNA in the donor cell into the assembling phage particles.

## Supplementary Material

Supplementary Data
