# Supplementary material for: Resistance gene transfer: induction of transducing phage by sub-inhibitory concentrations of antimicrobials is not correlated to induction of lytic phage
Source: J Antimicrob Chemother. 2017 Mar 20;72(6):1624–31. doi: 10.1093/jac/dkx056 (PMC5437526; doi:10.1093/jac/dkx056)
Supplement: Supplementary Data [file dkx056_Supp.docx]

**Supplementary data**

**Table S1. Genetic characteristics of the donor and recipient strains**

| strain | CC22 | *ermC* | *aac(6’)aph(2”)* | *Φ1* | *Φ2* | *Φ3* | *Φ 6* | *SaPI 4* | *qacA* | *rep_10_* | *tra* |
| --- | --- | --- | --- | --- | --- | --- | --- | --- | --- | --- | --- |
| 19A | **+** | **+** | **+** | **+** | **+** | **+** | **-** | **+** | **-** | **+** | **-** |
| 19B | **+** | **-** | **+** | **+** | **+** | **+** | **-** | **+** | **+** | **-** | **-** |

CC is clonal complex, *ermC* – gene encoding resistance to erythromycin, *aac*(6')-*aph*(2") gene encoding resistance to gentamicin, Φ1, Φ2, Φ3, Φ6 are positive for the phage integrase gene, SaPI4 is S. aureus pathogenicity island integrase, *rep*_10_  is plasmid based on the replication locus, *qacA* is putative antiseptic resistance gene, *tra* - *tra* gene cassette for conjugative transfer.

**Table S2. MIC of strains used in this study**

| **inoculum size CFU/ml** 1 X 10^6^ | | | |
| --- | --- | --- | --- |
| **MIC µg/ml**  **strain** | | | |
| **antibiotic** | **19A** | **19B** | **RN4220** |
| erythromycin | >256 | 0.5 | 0.25 |
| trimethoprim | 2 | 2 | 1 |
| ampicillin | >256 | >256 | 0.06 |
| mupirocin | 2 | 2 | 0.5 |
| tetracycline | 2 | 2 | 2 |
| novobiocin | 0.5 | 0.5 | 0.25 |
| cefoxitin | 64 | 64 | 0.5 |
| gentamicin | >256 | >256 | 1 |
| ciprofloxacin | >256 | >256 | 0.5 |

**Table S3. Primers for droplet digital PCR. Selected pairs of primers and probes used in ddPCR for the detection of A- Φ2 and ermC or B- Φ and nuc.**

A)

| **Target gene** | **Primer sequences**  **(5’ to 3’)** | **PCR amplicon size (bp)** | **dye** |
| --- | --- | --- | --- |
| Φ2 Fw | CTGGAAAACAAAATGTTGAAAAGA | 106 | n/a |
| Φ2 internal probe | TGGAATTCGAGCTATGTTGACAGAGGG |  | 5’FAM  3’ BHQ2 |
| Φ2 Rev | ACACCGGCGATTTGATTATT |  | n/a |
| *ermC* Fw | CGGATATAATACGCAAAATTGTTT | 110 | n/a |
| *ermC* internal probe | TCTTTTAGCAAACCCGTATTCCACGA |  | 5’Yakima Yellow  3’ BHQ2 |
| *ermC* Rev | TAATGCCAATGAGCGTTTTG |  | n/a |

B)

| *nuc Fw* | CCAACAGTATATAGTGCAACTTCAA | 120 | n/a |
| --- | --- | --- | --- |
| *nuc internal probe* | CCATCAATCGCTTTAATTAATGTCGCAG |  | 5’FAM  3’ BHQ2 |
| *nuc Rev* | TCTGAATGTCATTGGTTGACCT |  | n/a |
| Φ1 Fw | TTGTAGTCTGTCCAAACAAGAGC | 100 | n/a |
| Φ1 internal probe | CATAGCTTCGCCAATCCTTAAACCTGT |  | 5’Yakima Yellow  3’ BHQ2 |
| Φ1 Rev | AAGACATTGAAGAGCAAGATTACAGA |  | n/a |

Fw-forward, Rev-reverse, n/a-not applicable

**Table S4. The impact of sub-inhibitory antibiotics presented as transduction frequency (transductants per plaque forming unit). Data is from Figure 1.**

| Antibiotic | Transduction frequency (cfu/pfu) |
| --- | --- |
| control | 0 |
| 30ug/ml ampicillin | 1.94 x 10^-3^ |
| 30ug/ml gentamicin | 2.45 x 10^-2^ |
| 30ug/ml cefoxatin | 1.81 x 10^-2^ |
| 0.25ug/ml novobiocin | 3.67 x 10^-1^ |
| 30ug/ml ciprofloxacin | 2.47 x 10^-2^ |
| 1ug/ml tetracycline | 7.03 x 10^-1^ |
| 1ug/ml trimethoprim | 8.22 x 10^-3^ |
| 30ug/ml erythromycin | 6.63 x 10^-3^ |
| 1ug/ml mupirocin | 9.57 x 10^-1^ |

Supplementary

Figure S1.

**Figure S1.**  **Gentamicin and novobiocin combined with sub-MIC antibiotics can reduce lytic phage induction.** Combination of gentamicin (a, c) or novobiocin (b, d) with sub-inhibitory combinations of antibiotics, without (a, b) or with UV light (c, d). Combinations of antibiotics are indicated by hatching. Mean of at least three experiments in triplicate ± S.D, * p <. 0.05; ** p < 0.01, *** p< 0.001.

Figure S2

**Figure S2. Ratio of *ermC* to individual phage integrase genes packaged into phage particles, in response to induction by trimethoprim, ciprofloxacin and/or UV.** ddPCR was used to measure copy number, as in Figure 4.
